# Reduced neuronal size and mTOR pathway activity in the Mecp2 A140V Rett syndrome mouse model

**DOI:** 10.12688/f1000research.8156.1

**Published:** 2016-09-08

**Authors:** Sampathkumar Rangasamy, Shannon Olfers, Brittany Gerald, Alex Hilbert, Sean Svejda, Vinodh Narayanan

**Affiliations:** 1 Neurogenomics Division, Translational Genomics Research Institute, Phoenix, USA; 2 Center for Rare Childhood Disorders, Translational Genomics Research Institute, Phoenix, USA; 3 Barrow Neurological Institute, St.Joseph’s Hospital and Medical Center, Phoenix, USA; 4 School of Life Sciences, Arizona State University, Tempe, USA

**Keywords:** Rett syndrome, MECP2, Hippocampal neuronal cultures, Cerebellar granule neurons, Neuronal soma size, Neuronal nuclear size, mTOR pathway, IGF-1, Rictor

## Abstract

Rett syndrome (RTT) is a neurodevelopmental disorder caused by mutation in the X-linked *MECP2* gene, encoding methyl-CpG-binding protein 2. We have created a mouse model ( *Mecp2* A140V “knock-in” mutant) expressing the recurrent human *MECP2* A140V mutation linked to an X-linked mental retardation/Rett syndrome phenotype. Morphological analyses focused on quantifying soma and nucleus size were performed on primary hippocampus and cerebellum granule neuron (CGN) cultures from mutant ( *Mecp2* ^A140V/y^) and wild type ( *Mecp2* ^+/y^) male mice. Cultured hippocampus and cerebellar granule neurons from mutant animals were significantly smaller than neurons from wild type animals. We also examined soma size in hippocampus neurons from individual female transgenic mice that express both a mutant  (maternal allele) and a wild type *Mecp2* gene linked to an eGFP transgene (paternal allele). In cultures from such doubly heterozygous female mice, the size of neurons expressing the mutant (A140V) allele also showed a significant reduction compared to neurons expressing wild type MeCP2, supporting a cell-autonomous role for MeCP2 in neuronal development. IGF-1 (insulin growth factor-1) treatment of neuronal cells from *Mecp2* mutant mice rescued the soma size phenotype. We also found that *Mecp2* mutation leads to down-regulation of the mTOR signaling pathway, known to be involved in neuronal size regulation. Our results suggest that i) reduced neuronal size is an important *in vitro* cellular phenotype of *Mecp2* mutation in mice, and ii) MeCP2 might play a critical role in the maintenance of neuronal structure by modulation of the mTOR pathway. The definition of a quantifiable cellular phenotype supports using neuronal size as a biomarker in the development of a high-throughput, *in vitro* assay to screen for compounds that rescue small neuronal phenotype (“phenotypic assay”).

## Introduction

Rett syndrome (RTT) is a neurodevelopmental disorder caused by mutations in the X-linked *MECP2* gene encoding methyl-CpG binding protein 2 ^1^. Most human cases of *MECP2* mutation result in the classical form of RTT affecting predominantly girls ^2– 5^. Additionally, *MECP2* gene mutations have been linked to a broad range of other clinical and neurological phenotypes. One such mutation is *MECP2* A140V, seen in both male and female subjects with non-classic Rett phenotypes such as intellectual disability, parkinsonism, and neuropsychiatric symptoms ^6– 9^. The *MECP2* A140V mutation is a recurrent missense mutation (c.419C>T; p.Ala140Val) that shortens the alpha helix domain of the methyl CpG binding domain (MBD) without affecting methyl binding function. Protein functional studies have shown that this mutation instead results in the disruption of MECP2 interaction with ATRX (alpha thalassemia X-linked intellectual disability syndrome) ^10^. We have previously reported the characterization of a mouse model expressing the *Mecp2* A140V mutation ^11^. Our previous studies in hemizygous male mutants found (i) increased cell packing density, and (ii) aberrant dendrite branching, similar to pathological findings seen in human RTT and other neurodevelopmental disorders. Neuropathological studies in human RTT cases have shown a reduction in brain size, increased cell packing density, and smaller neuronal size (soma) ^12– 17^. Fine structure analysis of neurons in human RTT brain tissue revealed decreased dendritic arborization and spine density ^14, 17, 18^. The most common structural abnormalities reported in *Mecp2* mutant mouse models (including the *Mecp2*-null mice) are thinning of the cortical layers, reduction in neuronal soma size, and decreased dendritic complexity ^11, 19– 22^. Notably, neuronal soma size is considered a robust and reliable marker for MeCP2 function ^23^.

In the present study, we have focused on defining an *in vitro* neuronal phenotype using primary hippocampal and cerebellar granule neuron cultures from wild type and Mecp2 A140V male animals. Here we report results of a quantitative study examining neuronal soma size at different days *in vitro* (DIV), demonstrating that the neuronal size phenotype is a reliable marker of *Mecp2* mutant pathology. Given that RTT occurs predominantly in females, we were interested in evaluating an *in vitro* model of heterozygous females. Due to X-inactivation, neuronal cultures prepared from heterozygous females are a mixture of neurons expressing mutant MeCP2 and neurons expressing wild type MeCP2. We have developed a strategy to distinguish these two populations of neurons in cultures prepared from female *Mecp2*-mutation carriers and quantify soma size of mutant and wild type neurons. This approach allows us to compare wild type and mutant neurons plated on a single coverslip, prepared from a single female animal, eliminating confounding variables such as genetic background and culture conditions. Studies in such neuronal cultures from female heterozygotes allow us to differentiate between cell autonomous and cell non-autonomous effects of *Mecp2* mutation.

The molecular mechanisms by which *Mecp2* mutation results in a reduction of neuronal soma size are not well understood. Hippo and mammalian target of rapamycin (mTOR) pathways are considered to be primary molecular regulators of cell size ^24^. mTOR is a highly conserved serine/threonine protein kinase that participates in two distinct, multi-protein complexes, mTORC1, and mTORC2 ^25^. Rictor is an essential component of the mTORC2 (rapamycin-insensitive companion of mTOR) complex ^26^. In addition to the role of mTORC1, studies using rictor knockout cell model have identified unique functions for the mTORC2 in the maintenance of neuronal structure and function ^27^. Loss of rictor expression affects neuronal size (smaller soma size), morphology, and function ^28^. Likewise, the PI3K-AKT-mTOR signaling pathway has also been shown to regulate dendritic complexity, soma size, and spine morphology ^29^. Biochemical studies in *Mecp2*-mutant mice and stem cell model systems have found a significant reduction in mTOR signaling pathway activity ^30, 31^. In recent years, BDNF (brain-derived neurotrophic factor) and IGF-1 are pursued as therapeutic molecules in the treatment of RTT ^32– 34^. These molecules have been shown to rescue the normal neuronal size phenotype through mTOR pathway activation, thus supporting the idea that the mTOR pathway may play a significant role in RTT ^29, 34, 35^. To investigate the molecular mechanism of neuronal size reduction in *Mecp2* mutants, we analyzed the mTOR pathway in wild type and *Mecp2* A140V brain tissues. Here we report that downregulation of rictor and alteration of the mTOR signaling pathway in the *Mecp2* A140V brain represents a key element linking MeCP2 mutation to reduced neuronal size.

## Materials and methods

**Figure 1.**
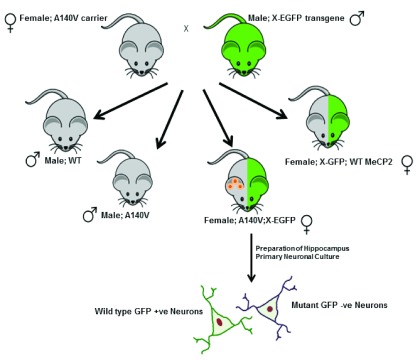
Breeding to generate female Mecp2 A140V:X-EGFP compound heterozygotes. Primary neuronal cultures were prepared from the female Mecp2 A140V X-EGFP compound heterozygotes (A140V:X-EGFP) for analyzing the neuronal soma size of mutant and wild type neurons in single coverslips.

**Animals:** The Institutional Animal Care and Use Committee (IACUC) of St. Joseph’s Hospital and Medical Center approved all animal experiments performed in this study (Protocol Approval #304 and Animal Welfare Assurance #A351-01). We have previously reported the construction and characterization of *Mecp2* A140V "knock-in" mice (B6N.129- *Mecp2* ^tm1.1Vnar/J^) used in this study ^11^. For the neuronal morphological studies, cell culture, and western blot experiments we utilized mutant A140V hemizygous males generated from our in house breeding protocol and control animals from C57BL/6NCrL background (Charles River Laboratories). We employed a novel strategy for generating heterozygous female mice in which we can distinguish neurons expressing wild type (WT) MeCP2 from those expressing the A140V mutant MeCP2 ( Figure 1). *Mecp2* A140V carrier females were crossed with male transgenic mice carrying the Enhanced Green Fluorescent Protein (X-EGFP) gene integrated into the X-chromosome (Tg (GFPX) 4 Nagy/J, Jackson Laboratory (USA)). All female offspring from such crosses express GFP in those cells in which the paternal X-chromosome is active. About 50% of the female animals generated in such a cross will be heterozygous for the A140V mutation (A140V: X-EGFP). In brain tissue and cultures prepared from these females, neurons expressing WT MeCP2 will be GFP-positive, while those expressing mutant MeCP2 will not.

**Cell culture:** Dissociated cultures of hippocampal neurons were prepared from control and mutant (A140V) male mice as well as from female transgenic mice (A140V: X-EGFP) at the postnatal age of 0–1 days (P0–P1) as described previously, with modifications ^36^. The hippocampi were dissected from the brain, and the tissues were enzymatically digested with 0.05% trypsin followed by mechanical dissociation using fire-polished Pasteur pipettes (trituration). The triturated suspension was allowed to settle for five minutes, and the supernatant was collected in a new tube and centrifuged for five mins at 200 X g to pellet the neurons. Final cell suspensions were prepared, counted, and plated on a poly-D-lysine coated glass cover slip. Hippocampal cultures were maintained in Neurobasal A with B-27 supplement without Insulin (Life Technologies, CA). To prevent clumping and allow for easy measurement of cell body size, we tested a range of seeding densities, ranging from 5,000–200,000 cells per coverslip in a 24-well plate. From our preliminary studies, we selected 50,000 cells per coverslip, and at selected days *in vitro*, coverslips containing cultured neurons were removed, and prepared for immunofluorescence after fixing with 4% paraformaldehyde. In some experiments, the cultures were treated with varying concentrations of IGF-1 (R&D Systems, Inc.) for 24 hrs. The cerebellar granule cells (CGN) were prepared from the postnatal age of 6 days (P6). The cerebellum was dissected from the brain, and the tissues were enzymatically digested with 0.05% trypsin followed by mechanical dissociation using fire-polished Pasteur pipettes. CGN cells were prepared and processed similarly to the hippocampus cell preparation. In the final step, the collected cells were left on a poly-D-lysine coated coverslip for 20 minutes in a humidified CO2 incubator (5% C02/95% air) at 37°C. The heavier cells tend to settle down on the coverslips while the small cerebellar granule neurons floated in the media. At the end of the incubation period, loosely adhered and floating granule neurons were dislodged, counted, and plated on poly-D-lysine coated glass cover with a seeding density of ~100,000 cells per coverslip in a 24-well plate.

**Immunostaining:** Mice were anesthetized and perfused transcardiac with saline followed by 4% buffered paraformaldehyde (PFA). The brains were removed and post-fixed in 4% PFA overnight at 4°C. Free-floating sections were used for immunohistochemistry. Coronal sections (40 µM) of the brain were prepared using a Vibratome 1000 (Vibratome 1000 plus; Jed Pella Inc.). The sections were stored at -20°C or at 4°C in PBS (phosphate buffered saline) containing 0.05% sodium azide until use. Immunostaining of the neuronal cells and the tissue sections were done following the established protocols. The tissue sections were permeabilized using 0.3% Triton-X in PBS for 30 minutes, blocked with 10% normal goat serum (NGS), followed by primary antibody staining in PBS containing 5% NGS overnight at 4°C. Neuronal cells on coverslips were fixed with 4% paraformaldehyde and sucrose in PBS for 15 minutes at room temperature and permeabilized with 0.2% triton-X 100 for 5 min. Tissues and the cells were stained with the primary antibodies, which included mouse anti-NeuN (1:1000 dilution), (Abcam; ab177487), Rabbit anti-Lamin B (1:1000 dilution) (Abcam; ab16048), Rabbit anti-GFP (1:500 dilution) (Millipore, AB3080)) and Rabbit/Mouse anti-beta-III tubulin (1:1000 dilution), (Abcam, ab18207) at 4°C overnight, and washed four times with PBS for every fifteen minutes. Appropriate secondary antibodies (1:2500 dilutions) such as Alexa Fluor 488 Goat Anti-Rabbit (Life Technologies, A11034) , Alexa Fluor 594 Goat Anti-Rabbit (Life Technologies, R37117), Alexa Flour 488 Goat Anti-Mouse (Life Technologies, A11029), and Alexa Flour 594 Goat Anti-Mouse (Life Technologies, A11005) were incuabtaed for two hours at room temperature and washed four times with PBS for every fifteen minutes. After primary and secondary antibody staining, the sections were counterstained with 4′-6-diamidino-2-phenylindole (DAPI), mounted with Prolong Gold mounting media (Invitrogen, Carlsbad, California), cover-slipped and stored at 4°C in the dark until imaged with a confocal laser-scanning microscope.

**Microscopy and size measurement:** To analyze neuronal morphology and quantify neuronal soma and nuclear size, we utilized a Zeiss LSM710 confocal laser-scanning microscope (Carl Zeiss, Germany). Confocal images were captured at 20X and 40X objective using the Zeiss microscope, and the soma size was quantified by tracing the outline of the neuronal cell body using beta-III tubulin staining. The cross-sectional area of selected neurons was measured using both Zen blue (Carl Zeiss, Germany) and Image J software (Version 1.48b) (NIH, Maryland, Bethesda). By dual staining with both DAPI/Lamin-b and TUJ1, we also measured the nucleus size of identified neurons. The nucleus cross-sectional area (nuclear size) in neuronal cells was determined by tracing the outline of the nucleus using Lamin-B or DAPI staining for the nuclear envelope, and DAPI was used to visualize nuclear DNA in the DAPI channel.

**Western blot:** Fresh brain tissues from wild type and mutant male animals were dissected and washed twice with ice-cold PBS. Tissue extracts were prepared according to the manufacturer’s instruction using N-PER (Neuronal Protein Extraction Reagent, Thermo Scientific, IL, USA) containing protease inhibitors II, III and a phosphatase inhibitor cocktail (Sigma-Aldrich, MO, USA). Supernatants were collected after centrifugation at 12,000 g for 20 minutes at 4°C, and protein concentrations were determined using BCA protein assay kit according to the manufacturer’s instruction (Thermo Scientific, IL, USA). Equal amounts of total protein (25–50 μg) from the samples were loaded and separated by PAGE electrophoresis using NuPAGE Novex Bis-Tris 4%–12% gels (Invitrogen) and transferred to PVDF membranes (Thermo Scientific, IL, USA). Primary antibodies against mTOR pathway molecules were all obtained from Cell Science Technology, MA, USA (Sampler Kit #9862, Kit #9864 Kit #9964) and beta III tubulin (ab52901) (Abcam, MA, USA) were used at a dilution of 1:1000 in TBST. Goat anti-rabbit IgG (H+L) was conjugated to DyLight™ 680 fluorescent dye from Cell Science Technology, MA, USA ( #5366) were used according to the manufacturer’s instruction to visualize the bands. Using an infrared imaging system (Odyssey; LICOR) the PVDF membranes were analyzed, and the signal intensity was determined with imaging software (Image Studio Lite 4.0 LICOR) and exported to a computer for graphic representation.

**Statistics:** For all quantitative measurements, statistical analyses of data were performed using GraphPad Prism 6 software. Data is reported as mean values, with error bars indicating standard error of mean. Variables with expected normal distributions, including quantitative measurements of neuronal soma and nuclear size, were tested by using the non-parametric two-tailed Mann–Whitney *U* test. For the frequency distributions, non-linear regressions with the best-fit values were utilized. Quantitative western blots in the biological replicates were analyzed using unpaired two-tailed t-test. *P* value of ≤0.05 is considered significant for all statistical tests.

## Results

Raw data for 'Reduced neuronal size and mTOR pathway activity in the Mecp2 A140V Rett syndrome mouse model' Click here for additional data file. Copyright: © 2016 Rangasamy S et al. 2016 Data associated with the article are available under the terms of the Creative Commons Zero "No rights reserved" data waiver (CC0 1.0 Public domain dedication).

### Neuronal soma size in male *Mecp2* A140V mutant mice, *in vitro*

**Figure 2.**
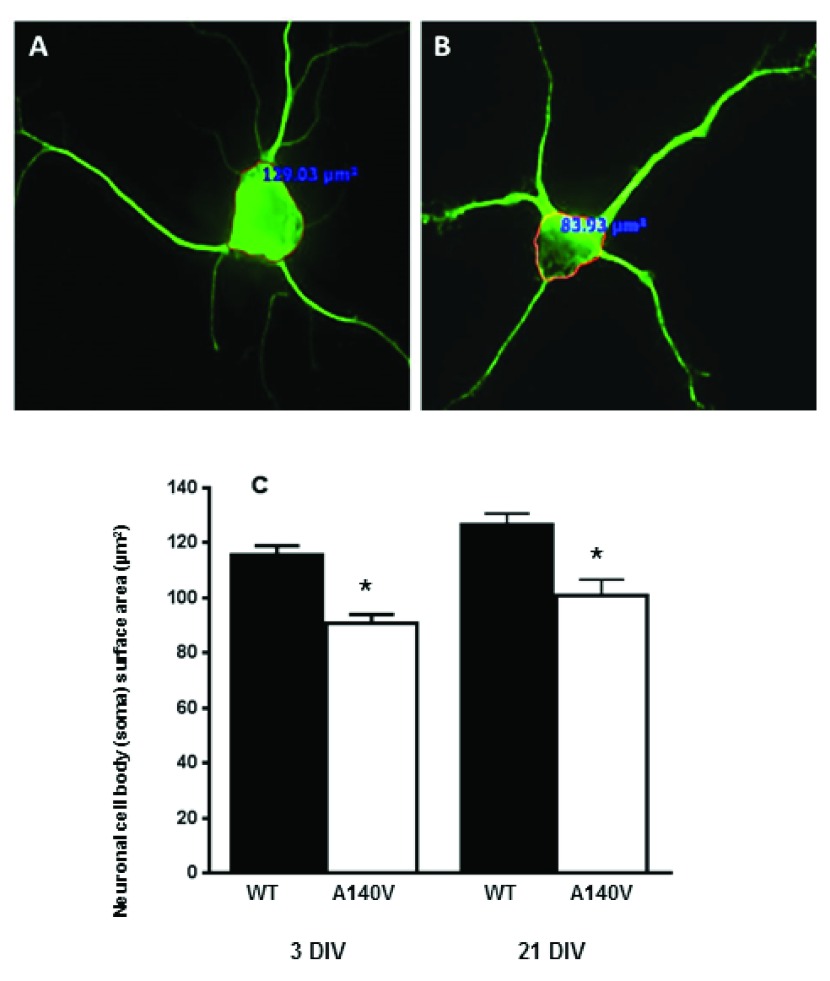
Soma size in cultured hippocampal neurons. Neurons were stained with antibodies against the neuron-specific marker beta-III Tubulin (Tuj-1). Soma size, defined as the cross-sectional area of an outline around the neuronal soma, was measured using image analysis software. Representative confocal images were obtained with 40 X objective. Immunofluorescence image showing: **A**) Wild type neuron, and **B**) MeCP2 A140V neuron. ( **C**) Quantification of neuronal soma size of wild type and mutant neurons at DIV 3 and 21 (Mean ± SEM, *, p <0.05, Student's t test).

Our laboratory has reported the construction and initial characterization of a mouse model expressing the *MeCP2* A140V mutation ^10^. We demonstrated increased cell packing density in the dentate gyrus, CA1, CA2, and CA3 of the hippocampus, frontal cortex, olfactory bulbs, and cerebellum in *Mecp2* A140V mice. Increased neuronal cell packing density in the postnatal brain appears to be a result of smaller soma size of mature neurons in Rett syndrome human brain ^12, 13, 16^. We examined the soma size of cultured primary hippocampus neurons from male *Mecp2* A140V (Mutant) and wild type animals. We chose to quantify neuronal soma size *in vitro*, as the definition of the soma boundary and measurement of soma size in tissues is much more difficult to perform in a consistent manner. Some intrinsic and extrinsic factors may affect neuronal soma size *in vitro*, such as variations in handling, cell seeding density, media pH, and composition, shrinkage during fixation. We prepared our cultures in a consistent manner to minimize variability, and compared samples of wild type and mutant cultures prepared and grown together. We used an immunofluorescence approach to analyze the soma size from cultured neuronal cells and measured cellular cross-sectional area (as a measure of soma size) at three days *in vitro* (DIV) and 21 DIV ( Figure 2A & B). We observed that the soma size of *Mecp2* A140V mutant neurons was significantly (p<0.01) reduced compared to wild type, at 3 DIV [116±3.2 µm ^2^; n=170 (Wt) vs 91±3.3 µm ^2^; n=151 (Mut)] and at 21 DIV [127±4.0 µm ^2^; n=40 (Wt) vs 101.0±5.6 µm ^2^; n=40 (Mut)] ( Figure 2C). These results indicate that the size reduction of mutant neuronal cells at both 3 and 21 DIV is comparable. It also suggests the presence of a key size regulatory mechanism for postnatal neurons and the disruption of this mechanism in RTT neurons.

### *In vitro* neuronal size distribution of *Mecp2* A140V mutant males

**Figure 3.**
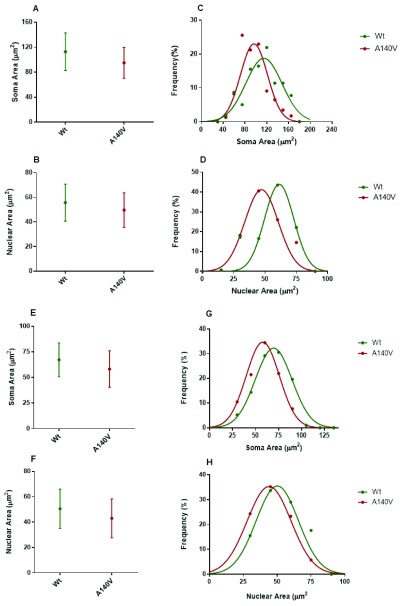
Neuronal soma and nuclear size distributions in the hippocampus and cerebellar granule cells. Average neuronal soma ( **A**) and nuclear ( **B**) size of hippocampus cells at 5 DIV from mutant and wild type. Frequency distributions of hippocampus soma ( **C**) and nuclear ( **D**) size are significantly different from mutant and wild type (p < 0.01). Average CGN neuronal soma ( **E**) and nuclear ( **F**) size at 5 DIV from mutant and wild type animals. Frequency distributions of CGN soma ( **D**) and nuclear ( **E**) areas are significantly different from mutant and wild type (p < 0.05).

We next analyzed the distributions of nuclear and soma size in the hippocampus and cerebellar granule cells at 5 DIV. As expected, comparison of mutant and wild type soma and nuclear sizes in these neurons also revealed a significant difference in mean size. The soma size of cultured hippocampus neurons at 5 DIV from the A140V males was significantly smaller than wild type ( Figure 3A). In addition to the established role of MeCP2 in the regulating neuronal soma size, the global chromatin modulating action of MeCP2 is also implicated in the regulation of postnatal neuronal nucleus size. Recent studies of embryonic stem cell (ESC)-derived neurons have shown a correlation between nucleus size and levels of MeCP2 expression ^37^. The nucleus size was measured as a surface area using DAPI and Lamin B staining. Quantification of nuclear size shows that mutant neurons were smaller when compared to wild type ( Figure 3B). We then examined the distributions of size in cultured hippocampus neurons and found that mutant neuronal soma and nucleus size were shifted towards smaller size area when compared to wild type neurons ( Figure 3C, D). We next elucidated the size of primary cerebellar granule neurons (CGN) from *Mecp2* A140V (Mutant) compared to wild type. CGN cultures offer the possibility of more homogenous cell culture compared to hippocampus neuronal culture, thus avoiding the influence of heterogeneity on size measurements. As expected, CGNs from mutant mice recapitulate the phenotype of smaller soma, and nucleus size observed in hippocampus neurons ( Figure 2). The CGN at 5 DIV from the male mutant brain were significantly lower while the nucleus size was also smaller when compared to wild type ( Figure 3E, F). Further, the distributions of mutant neuronal soma and nucleus size were shifted towards smaller size, when compared with wild type neurons, similar to the result with hippocampal cultures ( Figure 3G, H). From the distribution analysis, we could observe that the largest quartiles (bin) of soma and nucleus sizes in mutant neurons are significantly smaller than the largest quartile of the wild type neurons.

### Neuronal soma size in *Mecp2* A140V female heterozygotes

**Figure 4.**
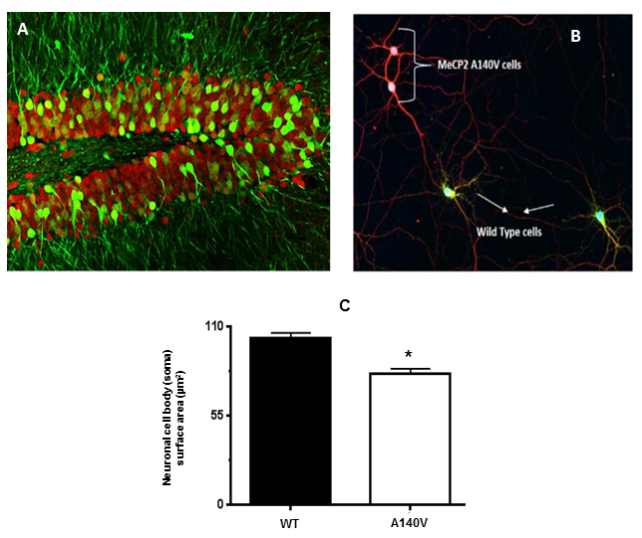
Neuronal soma size in heterozygous female A140V mice. ( **A**) Confocal image of the hippocampal dentate gyrus from a Mecp2 A140V: X-eGFP mouse brain coronal section. Staining with anti-NeuN (red) and anti-GFP (green) shows mosaicism. ( **B**) Hippocampal neurons from female Mecp2 A140V: X-eGFP mice were isolated and cultured in a single coverslip. Neurons were stained at DIV 3 with anti beta-III Tubulin (Tuj-1) (Red), and anti-GFP (Green) and the soma cross-sectional area was measured. MeCP2 A140V neurons do not express GFP and are stained by TuJ-1 alone (red) while that of wild type neurons are stained by both GFP and Tuj-1 (Greenish yellow). All the confocal images were captured with a 20X objective. ( **C**) Quantification of neuronal soma size of wild type and mutant neurons in these mosaic cultures (Mean ± SEM, *, p <0.05, Student's t test).

As described above, we generated female *Mecp2* A140V heterozygotes, which also carried an eGFP transgene on the wild type (paternal) X-chromosome ( Figure 1, Figure 4A). In hippocampal cultures prepared from such female mice (Mecp2 A140V; X-eGFP), neurons expressing the wild type MeCP2 allele (“WT neurons”) are GFP (+) while neurons expressing MeCP2 A140V (“mutant neurons”) are GFP (-) ( Figure 4B). After three DIV, coverslips were fixed and stained with mouse anti-beta-III tubulin antibody and rabbit anti-GFP antibody and counterstained with anti-rabbit 488 and anti-mouse 598. This strategy distinguished wild type (WT) neurons that are GFP positive (greenish yellow) from mutant (MUT) neurons that are GFP negative (red) in a single coverslip. We measured the size of MUT and WT neurons from such female cultures and found a significant reduction in soma size of mutant neurons [81.2±2.9 µm ^2^; n=135] compared to wild type [103±3.2 µm ^2^; n=137] ( Figure 4C).

### Rescue of neuronal soma size by IGF-1

**Figure 5.**
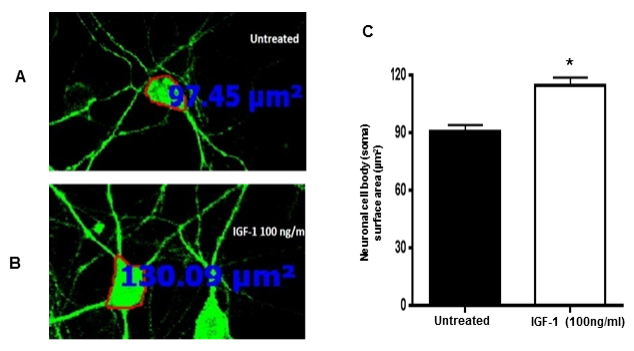
Treatment of neuronal cultures with IGF-1 rescues the small neuronal phenotype. Primary hippocampal cultures from Mecp2 A140V male animals were treated with IGF-1 (100 ng/ml) for 24 hrs on 3 DIV. Neurons were stained with anti-beta-III Tubulin (Tuj-1), and cross-sectional area of neurons was measured. Representative confocal images were obtained with 20 X objective. Immunofluorescence image showing ( **A**) untreated neurons and **B**) treated neurons with IGF-1. **C**) Quantification of neuronal soma size of mutant neurons without and with IGF-1 treatment revealed rescue of neuronal soma in the mutant neurons. (Mean ± SEM, *, p <0.05, Student's t-test).

IGF-1 is currently an attractive compound for the treatment of Rett syndrome. In preclinical studies, IGF-1 treatment of *Mecp2* mutant animals improves disease-related phenotypes ^33^. To investigate whether IGF-1 could reverse the soma size in mutant neurons, we treated the cultures of mutant hippocampus neuronal cells with IGF-1 at a concentration of 100 ng/ml for 24 hrs. Image analyses indicate that the recombinant IGF-1 treatment resulted in increased neuronal soma size of the mutant cells ( Figure 5A & B) [90.87±3.4 µm ^2^; n=119 (IGF-1 non-treated) vs 115.2±3.9 µm ^2^; n=116 (IGF-1 treated)] ( Figure 5C). The treatment of wild type neurons with IGF-1 also increased the cell size but did not reach significance.

### Alteration of mTOR pathway in *Mecp2* A140V mutant animals

**Figure 6.**
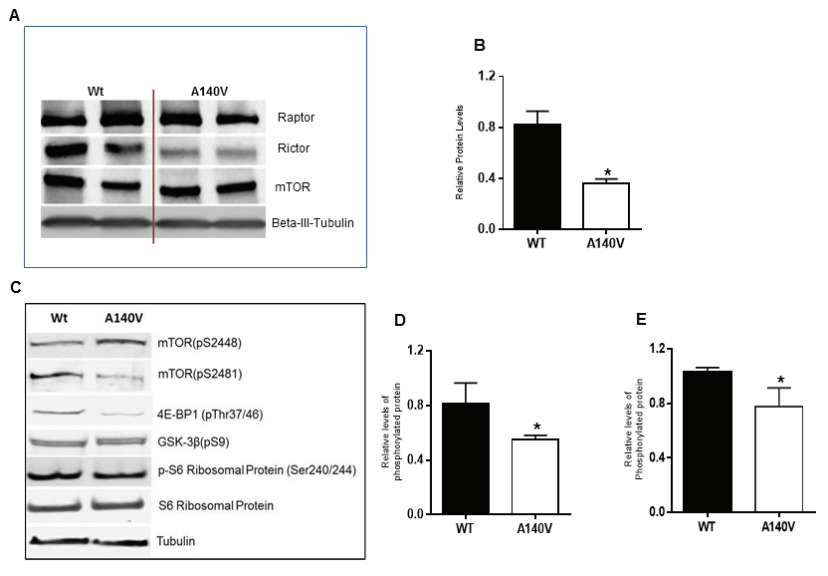
Alteration of the mTOR pathway in Mecp2 A140V mutant animals. **A**) Western blots of whole brain lysate from wild type and mutant mice were probed simultaneously with antibodies against rictor, raptor, and total mTOR. Tubulin was used as a loading control to normalize the protein expression, and the images were scanned and quantified for the protein levels. **B**) The graph shows a significant reduction in rictor protein level in mutant compared to control animals after normalization to beta-III tubulin levels (n=3 each) (Mean ± SEM, *p <0.05, Student’s t-test). **C**) Western blot analysis of brain lysate to detect phosphorylated mTOR (S2448 and S2481), phosphorylated S6 ribosomal protein, and 4E-BP1 and phospho GSK-3β levels in whole brain lysate of 16-week old mutant and wild type mice. To avoid interference in the detection of phosphorylated forms some of the proteins were detected on separate membranes. Using immunoblot, we assessed total and phosphorylated protein levels. Tubulin was used as an appropriate loading control in all panels. ( **D**) Phosphorylated mTOR (pS2481) and ( **E**) Phosphorylated 4E-BP1 protein levels in mutant compared to control animals (n=4 each) (Mean ± SEM, *p <0.05, Student’s t-test).

Several new lines of investigation indicate that the mTOR pathway is involved in the regulation of size in mammalian cells, including neurons. Details of the connection between the mTOR pathway and *MECP2* mutation are not clear, but a recent study has shown that AKT/mTOR pathway activity is reduced in *Mecp2* mutant neurons ^30, 31^. BDNF or IGF1 treatment of mutant animals activates the AKT/mTOR pathway and rescues neuronal soma size ^30^. Therefore, we examined mTOR pathway proteins in *Mecp2* A140V mutant and wild type animals by Western blot analysis. We compared the expression of major mTOR proteins including total mTOR, rictor, and raptor ( Figure 6A). Western blot analysis displayed a significant decrease (p<0.05) in the expression of rictor in mutant *Mecp2* A140V brain tissue ( Figure 6B) indicating that the mTORC2 pathway is altered in *Mecp2* A140V mutant mice. Given the role of mTORC2 as an upstream activator of AKT, this assumes greater significance. We also explored the phosphorylation of mTOR ( Figure 6C) and found that mTOR phosphorylation at S2448 ( Figure 6D), was not altered in mutant tissues, but observed a decrease in mTOR S2481 phosphorylation, which in turn was associated with a reduction in 4E-BP-1 phosphorylation ( Figure 6E) (p<0.05). We did not see changes in the phosphorylation status of S6 ribosomal protein, which may also reflect the status of p70 S6K1.

## Discussion

Rett syndrome is characterized by genotypic, phenotypic, and biological complexity in human subjects. MeCP2 has been shown to affect the expression of many genes and influences many distinct cellular processes in neurons and glia. This complexity poses a significant challenge in understanding the neurobiology of this disorder. Development of mouse models expressing human MeCP2 mutations has profoundly advanced our understanding of disease pathogenesis. The *Mecp2* A140V is one such mouse model developed in our laboratory and has been previously described to have cellular abnormalities similar to those seen in RTT ^11^. This is the first report of systematic and quantitative studies of soma and nucleus size in primary neuronal cultures from *Mecp2* mutant animals. In the present study, we have demonstrated a reduction in soma and nucleus size in cultured hippocampal neurons from hemizygous male *Mecp2* A140V mutant mice. The observed smaller soma size in *Mecp2* A140V neurons is consistent with that seen in other *Mecp2* mutant models ^19, 38– 42^. Studies from *Mecp2* ^−/ *y*^ *(Bird mice)* and *Mecp2 ^T158A^* ^/ *y*^ have demonstrated smaller neuronal size at early- and late-symptomatic time points ^38, 43^. Studies in *Mecp2*-null and Nestin-Cre *Mecp2* conditional mutants revealed that the size of the hippocampus, cerebral cortex and cerebellum were smaller compared to wild type; however, there were no differences in the brain architecture ^19^. Soma size of the hippocampal CA2 neurons in these mutant mice was found to be 15−25% less than that of the controls ^19, 43^. CamK-Cre mediated deletion of *Mecp2* gene in postnatal neurons also resulted in a smaller neuron phenotype, albeit less severe compared to that of germline or a Nestin-Cre mediated deletion of *Mecp2* ^19^. In the present study, soma size reduction in *Mecp2* A140V mutant neurons was in a similar range. Over-expression of MeCP2 in a human SH-SY5Y neuroblastoma cell line was shown to increase nucleus size, suggesting a general function for MeCP2 in nucleus organization. Our data indicates a significant reduction in nuclear size of mutant neurons, compared to wild type neurons. This observation is consistent with another recent study, which demonstrated that neurons generated from mouse embryonic stem cells (ESCs) lacking *Mecp2* had smaller nuclear size ^37^.

Previous studies have suggested both cell autonomous and non-autonomous effects of MeCP2 mutation ^40, 41, 44– 49^. However, these studies have not determined whether it is cell autonomous or non-autonomous function that contributes to distinct neuronal phenotypes in female *Mecp2* mutation heterozygotes. To investigate this problem in female mice, we devised an innovative approach utilizing heterozygous female carriers of A140V mutation, which also carried an X-linked GFP allele on the normal X-chromosome. In these animals, wild type neurons express GFP, while the neurons carrying mutant *Mecp2* (MUT neurons) do not express GFP. GFP-positive and GFP-negative neurons were resolved into different colors (green and red) by appropriate immunostaining. In hippocampal cultures prepared from such heterozygous *Mecp2* female carriers, we were able to differentiate between WT and MUT neurons plated on a single coverslip and quantified the size difference. The size of the MUT neurons from such female heterozygotes was similar to the neuronal size in cultures from hemizygous male *Mecp2* A140V animals. Thus, neither contact with wild type neurons nor the presence of factors secreted from wild type neurons rescued the morphological abnormalities in mutant neurons. Our results suggest that MeCP2 acts in a cell-autonomous manner in determining neuronal morphology.

IGF-1 is an emerging therapeutic agent for the treatment of RTT and related conditions. Treatment of MeCP2-null animals with IGF-1 peptide rescued morphological abnormalities ^33^. Further, recombinant human IGF1 (rhIGF1) treatment of *Mecp2* null mice resulted in improvement of physiological and behavioral symptoms ^50^. We have demonstrated that treatment of mutant neurons *in vitro* with recombinant IGF-1 restored the neuronal soma size almost to that of wild type neurons. Regulation of cell and organ size is mediated by numerous factors ranging from nutritional status to growth factors such as insulin, and insulin-like growth factors. Recent studies have shown that the PI3K–PTEN–AKT signaling pathway is critical in the regulation of neuronal soma size ^29^. Dysregulation of Akt/mTOR signaling and protein synthesis are demonstrated to be an important molecular feature in *Mecp2* mutant models and embryonic stem cell-derived neurons ^30, 31^. Our biochemical pathway analysis of the mTOR pathway indicates a significant reduction in the rictor protein level from *Mecp2* A140V mouse brain. New evidence suggests that rictor specifically affects the brain and neuronal size compared to other organs ^28^. Loss of rictor in the central nervous system (CNS) resulted in smaller neurons, most likely a cell-autonomous effect. We suggest that MeCP2-mediated regulation of rictor expression may be an essential link between MeCP2 and the mTOR pathway. Also, mTOR phosphorylation at S2448 –the mTOR variant associated with mTOR complex 1- was not altered, but there was an observed decrease in mTOR phosphorylation at S2481–the mTOR variant associated with mTOR complex 2- phosphorylation ^51^. The downstream signaling of mTOR pathway leads to activation of S6K1 and 4EBP1/eIF4E which independently regulates mammalian cell size through a translational mechanism ^52^. We found a reduction in 4E-BP1 phosphorylation in *Mecp2* A140V brain, independent of S6K1 activity. Decreased phosphorylation of 4E-BP1 has been shown to reduce cell size through downregulation of elF3 complex, independent of S6K1 activity ^53^. The transcriptional regulation of mTORC1 and mTORC2 components by MeCP2 is not known, but our results confirmed the dysregulation of mTORC2 signaling association with Mecp2 mutation. We thus suggest that the MeCP2 regulates mTOR pathway activity in neurons and mutation in *MECP2* results in downregulation of mTOR pathway that results in characteristic phenotypic feature of smaller neurons in Rett syndrome.

Here we show that *Mecp2* mutation in a mouse model directly affects the size of cultured neurons ( *in vitro*) across cerebellum and hippocampus neurons. Our data demonstrate the smaller neuronal size phenotype in primary neuronal cultures prepared from *Mecp2* mutant animals. We have also shown that IGF-1 treatment *in vitro* rescues this cellular phenotype and also report the down-regulation of mTORC1 and mTORC2- in *Mecp2* mutant brain tissues as a molecular correlate of the neuronal size phenotype. The present analysis was limited to measurement of cell size and its possible association with a molecular pathway that regulates cell size. Our study opens the possibility of a larger role for the mTORC1 and mTORC2 pathway in RTT, and we are currently examining its pathophysiologic meaning. The strong correlation between cell size and mTOR activity in MeCP2 models indicates that very basic cell biological pathways play a critical role in this characteristic cellular phenotype. We are now in a position to investigate the molecular pathways that connect MeCP2 mutation, mTOR down-regulation, decreased neuronal size, and aberrant neuronal function. Improved assays and tools (kinase assays, receptor activation, RNAi) based on molecular target-centric approaches for high-throughput screens is a common approach to improving translational success. However, a recent meta-analysis suggests that phenotype-based assays have greater success in FDA approval for first–in-class drugs ^54, 55^. For conditions such as RTT and autism, where there is a lack of targetable mechanisms, a phenotype-based assay may be the only practical approach for drug screening. Our work suggests that neuronal size may be a useful, quantitative, *in vitro* phenotypic marker for the development of high-throughput screening (HTS) assays to discover novel therapeutic agents. Repurposing of compounds already approved by the FDA for other uses, or discovery of new candidate compounds may progress quickly through smaller scale preclinical animal testing and then into clinical trials.

## Data availability

The data referenced by this article are under copyright with the following copyright statement: Copyright: © 2016 Rangasamy S et al.

Data associated with the article are available under the terms of the Creative Commons Zero "No rights reserved" data waiver (CC0 1.0 Public domain dedication). 

*F1000Research*: Dataset 1. Raw data for 'Reduced neuronal size and mTOR pathway activity in the Mecp2 A140V Rett syndrome mouse model', 10.5256/f1000research.8156.d134352 ^56^
