# Supplementary material for: Reduced neuronal size and mTOR pathway activity in the Mecp2 A140V Rett syndrome mouse model
Source: F1000Res. 2016 Sep 8;5:2269. [Version 1] doi: 10.12688/f1000research.8156.1 (PMC5040159; doi:10.12688/f1000research.8156.1)
Supplement: Peer review history

# Peer review: Referee response for version 1

**DOI:** 10.5256/f1000research.8773.r16241

**Published:** 2016-10-19

**Authors:** Ganeshwaran H. Mochida

**Affiliations:** 1 Division of Genetics and Genomics, Department of Medicine, Boston Children's Hospital, Boston, MA, USA

Rett syndrome is a neurodevelopmental disorder caused by mutations in the *MECP2* gene. The authors previously generated a knock-in mouse model of Rett syndrome ( *Mecp2* A140V). This paper describes *in vitro* studies of hippocampal and cerebellar granule neurons from the *Mecp2* A140V mice. The paper demonstrates that the soma and nuclear size of these neurons in the *Mecp2* A140V mice is decreased compared to the wild-type animals. The authors took advantage of a mouse line with an eGFP transgene on X chromosome to show that this size difference is a cell-autonomous effect. Further, they show that the small cell soma size phenotype can be rescued by IGF-1, and there are altered molecular signatures of the mTOR pathway in the mutant mouse brains. 

 It is a well-designed and well-executed study, and the data are clearly presented. This *in vitro* experimental model, with soma and nuclear size of neurons as biomarkers, could potentially be an attractive platform for high-throughput drug screening. In terms of the effect of A140V on the mTOR pathway, the reduction of Rictor, phosphorylated mTOR (S2481) and phosphorylated 4E-BP1 displayed on the Western blots is highly intriguing, but it would require further functional studies to link these molecular signatures to the phenotype of the A140V mutant neurons.

 Minor points:

- In Figure 5C, only the results of IGF-1 treatment in the mutant neurons are shown, but it would be better to also show the results of IGF-1 treatment in the wild-type neurons.
- In Figure 6A, there are two lanes of Western blots shown for each of wild-type and A140V. There should be an explanation as to what these two lanes represent.

I have read this submission. I believe that I have an appropriate level of expertise to confirm that it is of an acceptable scientific standard.

# Peer review: Referee response for version 1

**DOI:** 10.5256/f1000research.8773.r16174

**Published:** 2016-10-03

**Authors:** Subbiah Pugazhenthi

**Affiliations:** 1 Section of Endocrinology, Veterans Affairs Medical Center, Denver, CO, USA; 2 University of Colorado Anschutz Medical Campus, Aurora, CO, USA

Rett syndrome (RTT) is caused by mutations in *MECP2* gene which encodes methyl-CpG-binding protein 2. One such mutation is *MECP2* A140V. This group has previously generated a mouse model of RTT, expressing *Mecp2* A140V mutant. This mouse is extremely useful to characterize a complex disease like RTT, at the cellular and molecular level. This interesting study by Rangasamy *et al.,* focuses on the characterization of *in vitro* neuronal phenotype and reports several novel findings. Size distribution studies with hippocampal and cerebellar granule neurons suggest that the neurons from male mutant mice have smaller soma size. Following treatment with IGF-1, the neuronal soma size is rescued. This finding supports the potential therapeutic use of IGF-1 in RTT.

 The authors have also generated a novel female mouse model with heterozygous *Mecp2* A140V mutant, carrying X-linked GFP allele on the normal X-chromosome. By this approach, GFP-positive wild-type neurons and GFP-negative mutant neurons can be compared within the mixed neuronal populations. With this model, the authors demonstrate the cell-autonomous nature of MeCP2 action on the neuronal size. In addition, MeCP2 appears to play a role in the regulation of mTOR pathway. Defects in this pathway are observed in the mutant mice, as shown by decreases in the levels of phosphorylated mTOR (pS2481) and phosphorylated 4E-BP1. Overall, this is a well-designed study with significant findings that contribute to the understanding of a complex disease. Future studies are needed to further understand the mechanism linking mTOR down-regulation and neuronal dysfunction.

I have read this submission. I believe that I have an appropriate level of expertise to confirm that it is of an acceptable scientific standard.

# Peer review: Referee response for version 1

**DOI:** 10.5256/f1000research.8773.r16176

**Published:** 2016-09-27

**Authors:** Rajiv R. Ratan

**Affiliations:** 1 Sperling Center for Hemorrhagic Stroke Recovery, Burke Medical Research Institute, White Plains, NY, USA

The manuscript uses the experimental leverage of an *in vitro* cultures to document a change in soma size in neurons from a novel mouse mutant with a knock-in A140V mutation of the *MECP2* gene linked to the x-chromosome. Phenotype of hippocampal and cerebellar granule neurons from male and female mice were examined, and statistically significant reductions in soma size were noted. These reductions in soma size were associated with reductions in mTOR signaling and rescued by IGF-1, which drives mTOR signaling. The authors suggest that the reductions in neuronal size may be a good phenotype on which to screen for drugs or molecules that overcome MeCP2 mutant effects. 

 Overall this is a nice paper, with generally good controls and an intriguing message with clear clinical implications. Although not absolutely necessary, it would be nice to see if molecular or pharmacological inhibition of the PI3K-AKT-mTOR signaling pathway abrogated the effects of IGF1. It would also be useful to look at a growth factor that drives Erk signaling to see if it can overcome the effects. Finally forced expression of Rictor *in vitro* or *in vivo* might more directly link mTOR signaling with the Rett phenotype. Otherwise, a very interesting and important study.

I have read this submission. I believe that I have an appropriate level of expertise to confirm that it is of an acceptable scientific standard.

# Peer review: Referee response for version 1

**DOI:** 10.5256/f1000research.8773.r16351

**Published:** 2016-09-27

**Authors:** Lucas Pozzo-Miller, Wei Li

**Affiliations:** 1 Department of Neurobiology, University of Alabama at Birmingham, Birmingham, AL, USA

One of the cellular features of Rett syndrome replicated in MeCP2-based mouse models is a smaller cell body of neurons, which, together with the lack of neuronal cell death, results in a higher packing density of neurons in all brain regions studied to date. The study by Rangasamy *et al.* extends these observations to the *MECP2* A140V knock-in mouse model that was crossed with a GFP-based mouse reporter of X-chromosome inactivation. These mice, together with knock-in mice with a GFP sequence inserted into *Mecp2* gene, are a great tool to identify cells that express the mutant allele in the mosaic brain of heterozygous *Mecp2* female mice, allowing the characterization of cell autonomous and non-cell autonomous consequences of MeCP2 loss. The authors replicate the observation that the mTOR pathway is downregulated in *MECP2* A140V knock-in mice, as it is in *Mecp2* null mice. Finally, the authors describe that IGF-1 increased the cell body size of cultured neurons from *Mecp2 *A140V male mice. Overall, these observations confirm that neuronal cell body size is a reliable biomarker of MeCP2 dysfunction, that IGF-1 improves cellular features in a Rett model, and that *Mecp2* A140V knock-in mice replicate cellular features observed in Rett individuals and other Mecp2-based mouse models. The observations and approaches described by Rangasamy *et al.* represent a useful contribution to the field of translational and preclinical studies in Rett syndrome.

We have read this submission. We believe that we have an appropriate level of expertise to confirm that it is of an acceptable scientific standard.
